# Quantifying Heterogeneity in Host-Vector Contact: Tsetse (*Glossina swynnertoni* and *G*. *pallidipes*) Host Choice in Serengeti National Park, Tanzania

**DOI:** 10.1371/journal.pone.0161291

**Published:** 2016-10-05

**Authors:** Harriet Auty, Sarah Cleaveland, Imna Malele, Joseph Masoy, Tiziana Lembo, Paul Bessell, Stephen Torr, Kim Picozzi, Susan C. Welburn

**Affiliations:** 1 Epidemiology Research Unit, SRUC, Drummondhill, Inverness, United Kingdom; 2 Division of Pathway and Infections Medicine, Centre for Infectious Diseases, School of Biomedical Sciences, Edinburgh Medical School, The University of Edinburgh, Edinburgh, United Kingdom; 3 Boyd Orr Centre for Population and Ecosystem Health, Institute of Biodiversity, Animal Health and Comparative Medicine, College of Medical, Veterinary and Life Sciences, University of Glasgow, Glasgow, United Kingdom; 4 Tsetse & Trypanosomiasis Research Institute (TTRI), Tanga, Tanzania; 5 Serengeti Biodiversity Project, Tanzania Wildlife Research Institute, Arusha, Tanzania; 6 The Roslin Institute, The University of Edinburgh, Easter Bush, Midlothian, United Kingdom; 7 Department of Vector Biology, Liverpool School of Tropical Medicine, Liverpool, United Kingdom, Warwick Medical School, University of Warwick, Coventry, United Kingdom; University of Minnesota, UNITED STATES

## Abstract

**Background:**

Identifying hosts of blood-feeding insect vectors is crucial in understanding their role in disease transmission. Rhodesian human African trypanosomiasis (rHAT), also known as acute sleeping sickness is caused by *Trypanosoma brucei rhodesiense* and transmitted by tsetse flies. The disease is commonly associated with wilderness areas of east and southern Africa. Such areas hold a diverse range of species which form communities of hosts for disease maintenance. The relative importance of different wildlife hosts remains unclear. This study quantified tsetse feeding preferences in a wilderness area of great host species richness, Serengeti National Park, Tanzania, assessing tsetse feeding and host density contemporaneously.

**Methods:**

*Glossina swynnertoni* and *G*. *pallidipes* were collected from six study sites. Bloodmeal sources were identified through matching Cytochrome B sequences amplified from bloodmeals from recently fed flies to published sequences. Densities of large mammal species in each site were quantified, and feeding indices calculated to assess the relative selection or avoidance of each host species by tsetse.

**Results:**

The host species most commonly identified in *G*. *swynnertoni* bloodmeals, warthog (94/220), buffalo (48/220) and giraffe (46/220), were found at relatively low densities (3-11/km^2^) and fed on up to 15 times more frequently than expected by their relative density. Wildebeest, zebra, impala and Thomson’s gazelle, found at the highest densities, were never identified in bloodmeals. Commonly identified hosts for *G*. *pallidipes* were buffalo (26/46), giraffe (9/46) and elephant (5/46).

**Conclusions:**

This study is the first to quantify tsetse host range by molecular analysis of tsetse diet with simultaneous assessment of host density in a wilderness area. Although *G*. *swynnertoni* and *G*. *pallidipes* can feed on a range of species, they are highly selective. Many host species are rarely fed on, despite being present in areas where tsetse are abundant. These feeding patterns, along with the ability of key host species to maintain and transmit *T*. *b*. *rhodesiense*, drive the epidemiology of rHAT in wilderness areas.

## Introduction

For vector-borne diseases involving multiple host species, the contact rate between hosts and vectors is a key factor in determining the contribution of individual host species to the reservoir host community. Identifying host species in bloodmeals provides information about the host species fed on, but studies rarely report the density of available host species. Without being able to quantify the degree to which vectors are selecting or avoiding particular host species, it is difficult to fully understand vector behaviour and complex vector-host-pathogen dynamics.

Human African trypanosomiasis (HAT) is caused in East and Southern Africa by the hemoflagellate *Trypanosoma brucei rhodesiense* transmitted by species of tsetse fly (*Glossina* spp). Both domestic and wildlife host species play a role in HAT epidemiology [1,2]. *T*. *b*. *rhodesiense* has been identified in a number of wildlife species, including bushbuck (*Tragelaphus scriptus*), reedbuck (*Redunca redunca*), waterbuck (*Kobus ellipsiprymnus)*, hartebeest (*Alcephalus buselaphus*), warthog (*Phacochoerus africanus*), buffalo (*Syncerus caffer*), lion (*Panthera leo*) and hyena (*Crocuta crocuta*)[3–10]. Whilst it is clear that wildlife are important in maintenance and transmission of HAT, a lack of data has constrained any robust quantification of the dynamics between host, vector and pathogen. Wilderness areas have been identified as a priority for understanding HAT transmission, as they are likely to present a challenge in terms of future control [11].

In HAT, as in other multi-host vector-borne diseases, a range of species form a host community that maintains transmission, i.e. the HAT reservoir community [3,4]. However, the relative importance of different wildlife species in the overall transmission potential of the community is not well understood. This key gap limits our knowledge of how human disease risk might change, spatially or temporally, with different wildlife compositions or different host dynamics, and in turn how to mitigate or reduce risk of HAT to vulnerable communities. The relative importance of different wildlife species within the HAT reservoir community is dependent on both the ability of the species to maintain and transmit infection with *T*. *b*. *rhodesiense* and the rate of feeding of tsetse on the species. Therefore, as has been demonstrated for other vector-borne diseases [12,13], the host contact rates of tsetse are particularly important in determining the transmission potential of the community, and essential in understanding HAT maintenance and human disease risk.

Whilst a number of studies have focused on identification of host species in tsetse bloodmeals [14,15], almost none have assessed tsetse feeding preferences alongside host density. A notable exception is a study conducted in 1959 by Lamprey and others [16], which assessed host densities and analysed tsetse bloodmeals using serological methods, but was limited in study area and sample size. Whilst host density has been incorporated in studies for some vector-borne diseases, leading to good understanding of the dynamics of the system (for example for West Nile Disease [13], or Lyme Disease [17]), assessing vector feeding preferences without considering host density is not unusual in vector-host contact studies. Previous studies have indicated that tsetse are strongly selective, feeding predominantly on a small number of species, which has been linked to various ecological, physiological and behavioural reasons [18,19]. However, assessing tsetse feeding in the absence of information on host density leaves several key gaps. First, no information is gained on the hosts that are present, potentially in high densities, that are not fed on. Identifying these species is of value in further understanding the drivers that determine the diet of tsetse. Second, without knowing what other species are present, it is hard to make predictions about how feeding patterns might change, if host composition is altered. This is especially important if we wish to predict the effect of changes in host diversity, such as those associated with habitat fragmentation or declines of particular species, on disease incidence. Third, host density is a key parameter in development of models of disease dynamics in multi-host ecosystems, which is important in developing effective strategies for control, in this case to reduce human disease risk.

In the Serengeti National Park (SNP), Tanzania, savannah and woodland areas support large populations of the tsetse species *G*. *swynnertoni* and *G*. *pallidipes* [20,21] as well as numerous and diverse wildlife populations. Cases of HAT have been reported in this area for over one hundred years [22], with more recent cases in both the local population and tourists leading to continuing public health concerns[23,24]. Early bloodmeal studies in SNP using serological techniques identified warthog and buffalo as important hosts of *G*. *swynnertoni* [20,25]. More recently, sequence-based techniques have proved successful at identifying hosts of *G*. *pallidipes* and *G*. *swynnertoni* [26]. Previous serological-based techniques required antiserum to be raised against each species likely to be present, which was a significant obstacle to identifying unexpected or unusual host species and often meant hosts could not be identified to a species level. These new approaches, combined with analysis of host species densities, provide an exciting opportunity to refine previous findings.

The aims of this study were to assess the contribution of different wildlife host species to the diet of *G*. *pallidipes* and *G*. *swynnertoni* in SNP using sequence-based methods, and to quantify the degree of host selection and avoidance of *G*. *swynnertoni* and *G*. *pallidipes* by comparing bloodmeal sources with the relative densities of wildlife host species.

## Methodology

### Study Site

**Fig 1:**
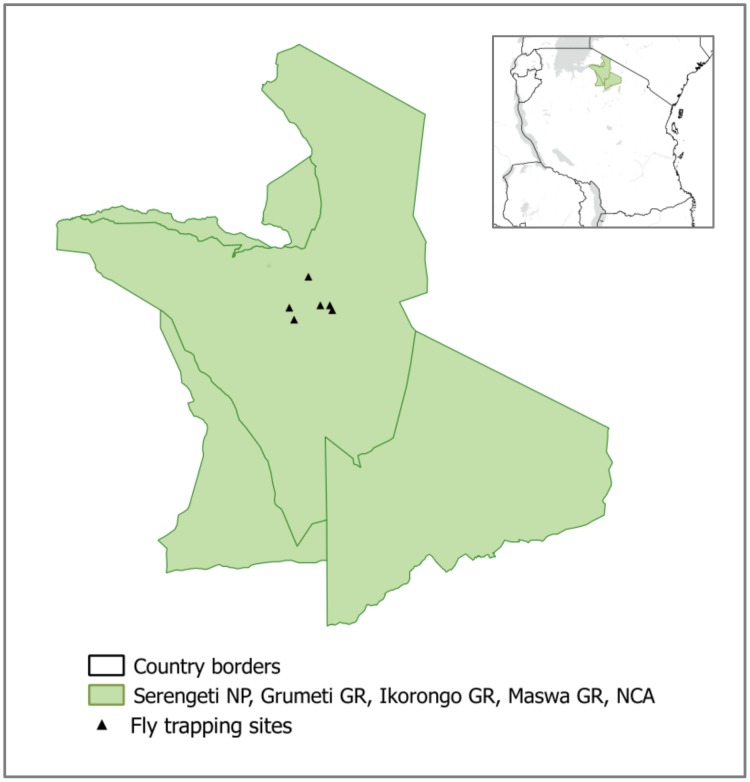
Map of study sites in Serengeti National Park, Tanzania. The map shows an outline of the protected area boundaries of Serengeti National Park (SNP), Grumeti, Ikorongo and Maswa Game Reserves (GR) and Ngorongoro Conservation Area (NCA), within Northern Tanzania.

All activities were approved by the Tanzania Wildlife Research Institute, Tanzania National Parks and Tanzania Commission for Science and Technology (permit numbers 2005–102-CC-2005-07, 2006-143-ER-2005-07, 2007-163-ER-2005-07). Sample sites in SNP were stratified by vegetation type in order to obtain variety in wildlife host density and composition. Using the grid analyst extension in ArcView GIS 3.2 (ESRI), a 1km^2^ grid was overlaid on the map, extending in a circle with radius 20km and the centre in Seronera, where fly processing was carried out (Fig 1). Each square was classified by the predominant vegetation type(s): grassland; savannah; open woodland; or dense woodland. For one type to be classified as predominant, it comprised over 90% of the pixels in the grid square. For two predominant types, each type comprised more than 30% of the square, with no other type more than 10%. A buffer was added to select only grid squares within 1km from a road, to allow quick transportation of flies back to the laboratory in Seronera. Although the proximity to roads may introduce bias into the sampling, it was logistically impossible to repeatedly visit sites less accessible than this. Two grid squares were randomly selected in each of the following vegetation types using a random number generator to give a total of six study sites: savannah, open woodland, and mixed savannah and open woodland. No sampling was conducted in thick woodland because vehicle access was not possible. No sampling was conducted in grassland areas as pilot sampling indicated too few tsetse would be caught for meaningful analysis.

### Bloodmeal collection

The collection of tsetse bloodmeals was part of a study in which tsetse were dissected to look for the presence of trypanosomes [21]. Tsetse sampling was conducted between August and October 2006 in collaboration with the Tsetse and Trypanosomiasis Research Institute, Tanga, Tanzania. In each site, three Epsilon traps [27] were installed for between five and eleven days, depending on trap catches. Each trap was situated at least 200m from the next, erected in mottled shade to reduce fly mortality, baited with 4-methylphenol (1 mg/h), 3-n-propylphenol (0.1 mg/), 1-octen-3-ol (0.5 mg/h) and acetone (100 mg/h)[28]and emptied twice daily. For any flies with evident bloodmeals, the midgut was dissected out and smeared onto one circle of a FTA Classic Card (Whatman) using the edge of a microscope slide. Smears were allowed to dry then stored at room temperature in foil envelopes with desiccant. The species and sex of the fly was recorded.

### Wildlife density

The density of large mammal species in each study site was estimated using data on wildlife observations recorded from driving line transects and analysed using Distance software [29].This method has been considered the most appropriate for assessing density of large mammal species occurring at low to moderate densities in Maasai Mara in Kenya, which is part of the Serengeti Ecosystem [30]. Three parallel 3km transects were driven in each study site, centred on the grid square. In some areas, geographical features (such as rivers or thick bush) meant it was not possible to drive the entire transect length, so reduced transects were used. Transects were established using a hand held GPS (Garmin) to follow a grid line and driven by vehicle. With one exception where woodland was otherwise too thick to penetrate (part of a transect in one study site only), they did not follow roads. Each transect was repeated twice monthly between December 2005 and July 2007 between 07.00 and 10.00. Whenever animals were observed, the perpendicular distance to the centre of the group was measured using a range finder and the species and number of animals in the group was confirmed using binoculars and recorded.

The data indicated that the density of wildebeest, zebra and Thomson's gazelle (see S1 Table for scientific names) in each study site increases acutely for some months, associated with seasonal migration [31]. These months did not coincide with the months when the tsetse bloodmeal samples were collected. Therefore, months when large numbers for these migrating species were present were excluded from the analysis.

Exact distances were used for density analysis. Histograms of the distance observations were examined to check for obvious violations of the assumptions. The distance data were truncated to remove the highest distances, which can be difficult to model; 5–10% of distance observations were truncated depending on the species [32]. For each species, the detection function was modelled using the half-normal, uniform and hazard rate functions provided in Distance and the function chosen which best fitted the data, based on Akaike information criterion (AIC), goodness of fit tests, biological plausibility and the shape of the data histogram [32]. Cosine, simple and hermite polynomial adjustments were added sequentially where necessary, based on the corrected AIC. Global detection functions were used in preference to study site specific detection functions, as for the majority of cases the summed AIC of each stratum detection function was higher than the AIC from global detection functions [32]. Exact cluster sizes were used. The logarithm of cluster size was regressed against the detection probability to correct for any size distance bias, unless the regression was not significant at 0.15, in which case the mean cluster size was used for density estimation [32].Cluster size was estimated per study site when sufficient observations were present for each site, or it was biologically plausible that the cluster size rate could differ between sites; otherwise mean cluster size was used.

Since the transects were used only to assess the density within the 1km grid square of interest, the variance in the spatial variability of the encounter rate was not included in the variance estimates, but only the variance in cluster size and detection probability [29].Confidence intervals were calculated in Distance using a Poisson model with over-dispersion set to 0 [29].

### Bloodmeal analysis

DNA was eluted from the FTA cards for amplification as follows: two 2mm discs were cut out of each FTA card sample, discs were washed for two 15 minute washes with FTA wash, followed by two 15 minute washes with 1xTE buffer. Discs were dried at 37°C for 30 minutes. 50μl of 5% (w/v) chelex suspension was added to each tube, and tubes were incubated for 30 minutes at 90°C [33].

Bloodmeals were identified following the protocols described by Muturi et al. [26]. Bloodmeal hosts were identified by amplification of a 359bp fragment of the mitochondrial cytochrome b gene, using primers Cb1 and Cb2. PCR amplification was carried out in 25μl reactions containing 5μl Supertaq PCR buffer (HT Biotechnologies, Cambridge, UK) (10mM TrisHCl, 50mM KCl, 1.5mM MgCl2, pH8.3), 1μM of each primer (synthesized by Integrated DNA Technologies), 800μM total dNTPs, 0.7IU of Biotaq Red DNA polymerase (Bioline Ltd, London, UK) and 1 μl of eluted DNA. PCR was carried out in a Dyad Peltier thermal cycler under the following conditions: 95°C for 10 minutes, 35 cycles of 94°C for 30 seconds, 52°C for 30 seconds, 72°C for 45 seconds, followed by a final extension of 72°C for 5 minutes. Products were visualised on a 2% (w/v) agarose gel. If a clear band was present, it was excised and the DNA extracted using a QiagenMinelute kit following the manufacturer’s instructions (Qiagen). Extracted DNA was submitted for bidirectional sequencing using the amplification primers (GATC Biotech).

### Sequence analysis

Sequence quality was assessed visually in Bioedit [34]. Forward and backward sequences were aligned and consensus sequences created. Two methods of species identification were used. First, sequences were compared to published sequences in the NCBI database using BLAST (Megablast); those that showed a clear match with existing sequences and a shared similarity of 97% or greater were assigned a species identification. Second, reference sequences were identified in Genbank for species likely to be present, and a reference database created. Species included were all larger mammal species [35], along with four small rodent species, four reptile species and three bird species. Sequences were available for all species except *Dendrohyrax arboreus* (tree hyrax) for which the closely related *Dendrohyrax dorsalis* (Western tree hyrax) was used instead. Reference sequences for *Otocyon megalotis* and *Galarella sanguinea* could not be aligned and were excluded. Reference sequences for *Heterohyrax brucei* and *Bitis arietans* did not cover the entire sequence length but were still included in the alignment. Species included in the reference database and accession numbers of reference sequences used are provided as supporting information, and are listed in S1 Table. All blood meal sequences of sufficient quality to align were included in an alignment with the reference database using the ClustalW [36] accessory application in Bioedit. A neighbour-joining tree was constructed using Geneious [37] under a Hasegawa-Kishino-Yano (HKY) [38] model of substitution. Species identifications were assigned if a sequence clearly clustered within a group with a reference species. Sequences that did not definitively sit within a group were not assigned a species identification. A binomial logistic regression was used to look for significant differences in bloodmeal identification rate between tsetse species, sex or study site.

### Analysis of feeding indices

Feeding indices were calculated using methods based on forage ratios, as described previously [13,39,40],

$$w_{i}=\frac{proportion\;of\;host\;species\;i\;in\;blood\;meals}{proportion\;of\;host\;species\;i\;in\;environment}=\frac{o_{i}}{p_{i}}$$

Where *o*_*i*_ was the proportion of bloodmeals from species *i* out of the total bloodmeals for each study site and *p*_*i*_ was the density of species *i* out of the total density of the species identified in each study site. Values of *w*_*i*_ above 1 indicate hosts are selected more frequently than would be expected through random selection. Values of *w*_*i*_ between 0 and -1 indicate hosts are avoided. Some species were present in a study site but never identified in a bloodmeal. These were given the bloodmeal value of 0.5 as described previously [13]. If this resulted in a feeding index above 1 (which arose if the density was very low), the bloodmeal value was set at 0, i.e. no preference or avoidance was expressed. This conservative measure meant that a number of species were not assigned a feeding index value, but avoided giving artificially inflated and meaningless values. Some hosts were identified in bloodmeals, but were not detected by transect surveys, i.e. the density was below the level of detection. These species were given the same density as the lowest density found for any species [39],which was 0.2/km^2^.

To test whether the feeding index, *w*_*i*_, was significantly different from 1, *P* values were calculated based on 10000 simulations from a multinomial distribution to compare the observed frequency of feeding for each species with the null hypothesis (the expected frequency if feeding occurred in proportion to the density) for each study site (as in [41]). *P* values less than 0.05 were considered significant.

All statistical analyses were conducted in R (www.R-project.org).

## Results

### Bloodmeal analysis

Bloodmeals were obtained from 304 *G*. *swynnertoni* and 89 *G*. *pallidipes*. For *G*. *swynnertoni*, 244 samples generated PCR products for sequencing. Of these, 205 sequences matched published sequences in Genbank on BLAST search with 97% similarity or higher (67% of the bloodmeal samples). Out of the 244, 18 sequences were not of sufficient length or quality to include in an alignment; the remaining 222 sequences were aligned for cluster analysis. By cluster analysis, 220 were identified (72% of samples). Two sequences were included in the alignment but did not cluster with any reference sequence or each other. From the chromatograms, these were both observed to be poor quality sequences. Sixty-five samples from *G*. *pallidipes* generated PCR products for sequencing. Of these, 36 were identified using the BLAST approach (40% of samples). Nineteen were not of sufficient length or quality to include in an alignment. Of the 46 aligned for cluster analysis, all were identified (52% of samples). For both *G*. *swynnertoni* and *G*. *pallidipes*, the cluster analysis confirmed all identifications by BLAST searching. Additional samples identified by cluster analysis were distributed across the commonly identified species, and did not alter the general patterns obtained, for example they did not suggest that particular species or groups of species had been systematically non-identified by the BLAST method. Therefore, the results of the cluster analysis method were used for further analyses, since it provided a better identification rate. The difference in the proportion of samples successfully identified between *G*. *swynnertoni* and *G*. *pallidipes* was significant (p<0.001, χ^2^_1_ = 12.9). There was no difference in identification success by sex (p = 0.37, χ^2^_1_ = 0.80) or study site (p = 0.089, χ^2^_5_ = 9.55) when these were included in a logistic regression analysis alongside tsetse species. Genbank accession numbers for the blood meal samples that were matched to >97% in BLAST searches are listed in S2 Table.

**Fig 2:**
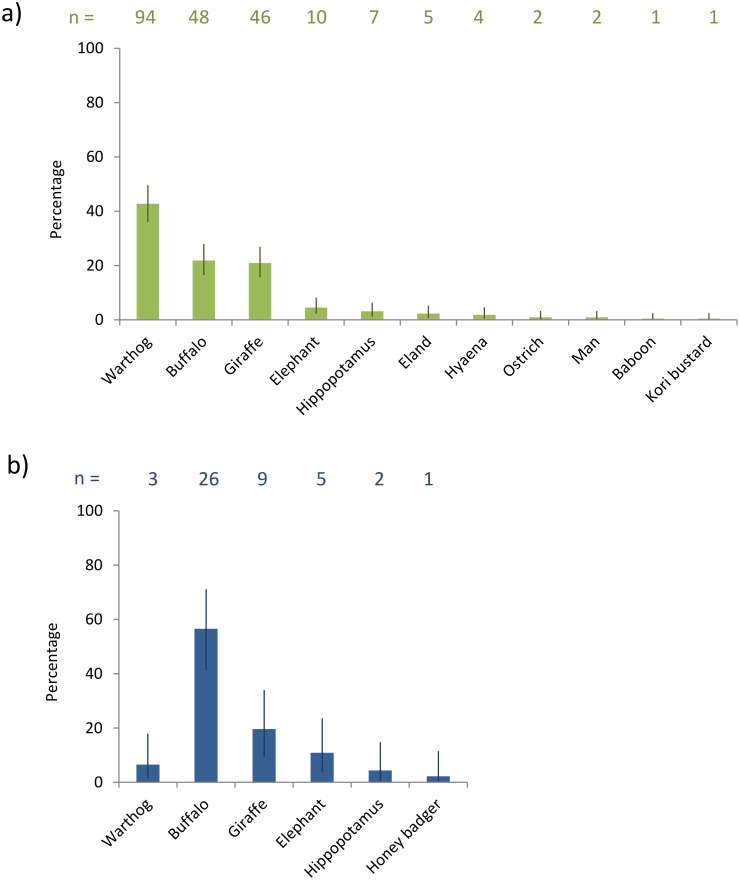
Wildlife hosts identified in bloodmeal samples. The graphs show the percentage of bloodmeals identified per species, out of the total identified samples for (a) *Glossina swynnertoni* and (b) *G*. *pallidipes*. The number of samples identified as each species is shown (n), out of a total of 220 and 46 samples identified for *G*. *swynnertoni* and *G*. *pallidipes*, respectively. Error bars show 95% binomial confidence intervals.

The proportions of bloodmeals identified as each wildlife species are shown in Fig 2. For *G*. *swynnertoni*, warthog, buffalo and giraffe made up a large proportion of bloodmeals (combined total of 85% of bloodmeals identified) with warthog being the most commonly identified (43% of bloodmeals identified). For *G*. *pallidipes*, buffalo DNA was most common (57%), with giraffe and elephant also important (combined total of 83% of bloodmeals identified). In three study sites, two or less *G*. *pallidipes* were found. In the three sites where both tsetse species found, the proportion of blood meals from *G*. *swynnertoni* were giraffe (39%), warthog (26%) and buffalo (17%). The proportion of bloodmeals derived from each wildlife species, for each study site, is shown in Table 1. There were no statistically significant differences in the hosts identified from male and female tsetse bloodmeals.

**Table 1:** Distribution of tsetse bloodmeals identified by host species.

	*Glossina swynnertoni*												*Glossina pallidipes*											
Study site	1		2		3		4		5		6		1		2		3		4		5		6	
	n	%	n	%	n	%	n	%	n	%	n	%	n	%	n	%	n	%	n	%	n	%	n	%
Baboon	0	0	0	0	1	1.9	0	0	0	0	0	0	0	0	0	0	0	0	0	0	0	0	0	0
Birds	0	0	3	6.7	0	0	0	0	0	0	0	0	0	0	0	0	0	0	0	0	0	0	0	0
Buffalo	0	0	13	24.5	2	3.8	21	51.2	10	66.7	2	11.1	2	14.3	0	0	0	0	1	50	17	94.4	6	54.5
Dikdik	0	0	0	0	0	0	0	0	0	0	0	0	0	0	0	0	0	0	0	0	0	0	0	0
Eland	0	0	0	0	4	7.7	1	2.4	0	0	0	0	0	0	0	0	0	0	0	0	0	0	0	0
Elephant	7	17.1	0	0	0	0	2	4.9	1	6.7	0	0	4	28.6	0	0	0	0	0	0	1	5.6	0	0
Giraffe	18	43.9	3	5.7	6	11.5	10	24.4	2	13.3	7	38.9	7	50	0	0	0	0	1	50	0	0	1	9.1
Grant's gazelle	0	0	0	0	0	0	0	0	0	0	0	0	0	0	0	0	0	0	0	0	0	0	0	0
Hartebeest	0	0	0	0	0	0	0	0	0	0	0	0	0	0	0	0	0	0	0	0	0	0	0	0
Hippopotamus	0	0	0	0	0	0	0	0	0	0	7	38.9	0	0	0	0	0	0	0	0	0	0	2	18.2
Hyaena	1	2.4	0	0	2	3.8	1	2.4	0	0	0	0	0	0	0	0	0	0	0	0	0	0	0	0
Impala	0	0	0	0	0	0	0	0	0	0	0	0	0	0	0	0	0	0	0	0	0	0	0	0
Large felids	0	0	0	0	0	0	0	0	0	0	0	0	0	0	0	0	0	0	0	0	0	0	0	0
Reedbuck	0	0	0	0	0	0	0	0	0	0	0	0	0	0	0	0	0	0	0	0	0	0	0	0
Small canids	0	0	0	0	0	0	0	0	0	0	0	0	0	0	0	0	0	0	0	0	0	0	0	0
Thomson's gazelle	0	0	0	0	0	0	0	0	0	0	0	0	0	0	0	0	0	0	0	0	0	0	0	0
Topi	0	0	0	0	0	0	0	0	0	0	0	0	0	0	0	0	0	0	0	0	0	0	0	0
Warthog	14	34.1	33	62.3	37	71.2	6	14.6	2	13.3	2	11.1	1	7.1	1	100	0	0	0	0	0	0	1	9.1
Wildebeest	0	0	0	0	0	0	0	0	0	0	0	0	0	0	0	0	0	0	0	0	0	0	0	0
Zebra	0	0	0	0	0	0	0	0	0	0	0	0	0	0	0	0	0	0	0	0	0	0	0	0
Other	1	2.4	1	1.9	0	0	0	0	0	0	0	0	0	0	0	0	0	0	0	0	0	0	1	9.1
**Total**	**41**		**53**		**52**		**41**		**15**		**18**		**14**		**1**		**0**		**2**		**18**		**11**	

Number (n) of bloodmeal samples identified as each species, for each of six study sites, and the proportion (%) out of total identified samples for a) *Glossina swynnertoni* and b) *G*. *pallidipes*. Denser colour indicates more blood meals identified.

### Wildlife Density

In total, over 3400 individual observations were recorded (one observation refers to either a group of animals or an individual animal). During the study period, transects were driven in each study site between 34 and 36 times. In May 2006 and May 2007 access was impossible due to the long rains, and in August 2006 one or two transects were missed in five study sites due to logistical constraints.

For 12 species, the number of observations recorded was sufficient to estimate the density in each study site (Tables 2 and 3). For baboon, elephant, and reedbuck the numbers of sightings were sufficiently low (between 40 and 70 after truncation) such that global densities were used rather than calculating a density value for each study site. Bat-eared foxes and black-backed jackals were analysed together to give a global density for small canids. Lions, cheetah and leopard were analysed together to give a global density for large felids. In addition, a number of other species were seen but the number of observations was too low to assess density (<40) so these species were excluded from analysis. Species densities by study site are shown in Table 4.

**Table 2:** Models chosen for distance analysis of 17 wildlife species or groups of species most commonly sighted during transects.

Species	Number of observations after truncation	Detection function model	Cluster size estimate		Density estimate
			Global or Stratum level	Mean or regression	Global or Stratum level
Baboon	40	half normal	global	mean	global
Buffalo	253	half normal	stratum	regression	stratum
Dikdik	93	hazard	global	mean	stratum
Elephant	55	half normal	global	mean	global
Giraffe	197	uniform (2^nd^ order cosine)	stratum	mean	stratum
Grant's gazelle	92	uniform (1^st^ order cosine)	stratum	mean	stratum
Hartebeest	160	half normal	stratum	mean	stratum
Hyaena	95	hazard	global	mean	stratum
Impala	440	half normal	stratum	mean	stratum
Large felids	40	hazard	global	mean	global
Reedbuck	43	half normal	global	mean	global
Small canids	66	hazard	global	mean	global
Thomson’s gazelle	426	half normal	stratum	regression	stratum
Topi	119	half normal	stratum	mean	stratum
Warthog	249	hazard	stratum	mean	stratum
Wildebeest	71	half normal	stratum	regression	stratum
Zebra	235	half normal	stratum	mean	stratum

**Table 3:** Density of wildlife host species in six study sites.

Species	Density by study site (animals per km^2^)					
	1	2	3	4	5	6
Baboon	2.4 (1.7–3.4)	2.6 (1.8–3.7)	2.6 (1.8–3.7)	2.6 (1.8–3.7)	2.6 (1.8–3.7)	2.6 (1.8–3.7)
Buffalo	7.1 (2.7–19)	9.1 (3.9–21.9)	4.4 (3.0–6.4)	7.1 (5.2–9.7)	8.1 (6.4–10.1)	10.9 (8.5–14.0)
Dikdik	0	1.8 (1.4–2.3)	2 (1.6–2.7)	3.2 (2.5–4.2)	4.6 (3.5–6.1)	15.3 (11.6–20.0)
Elephant	2.4 (1.9–3.2)	2.4 (1.9–3.2)	2.4 (1.9–3.2)	2.4 (1.9–3.2)	2.4 (1.9–3.2)	2.4 (1.9–3.2)
Giraffe	2.5 (1.7–3.7)	3.1 (2.5–4.0)	4.7 (3.6–6.2)	2.7 (2.1–3.6)	6.3 (5.3–7.5)	5.1 (4.0–6.4)
Grant's gazelle	2.4 (1.8–3.3)	4.2 (3.3–5.3)	2.7 (1.6–4.4)	0.09 (0.03–0.26)	0.3 (0.08–1.1)	0
Hartebeest	1.5 (0.96–2.5)	5.4 (4.3–6.8)	7 (5.2–9.5)	3.4 (2.4–4.8)	1.1 (0.63–1.8)	0.24 (0.0001–399)
Hyaena	5.4 (3.6–8.1)	1.5 (1.0–2.2)	1.5 (1.0–2.3)	1.1 (0.73–1.7)	0.29 (0.19–0.43)	0.9 (0.6–1.3)
Impala	1.8 (0.72–4.4)	22.5 (18.0–28.2)	47.6 (36.9–61.4)	35.7 (28.7–44.5)	47.4 (39.9–56.3)	83 (68.6–100.4)
Large felids	1.4 (0.78–2.6)	1.4 (0.78–2.6)	1.4 (0.78–2.6)	1.4 (0.78–2.6)	1.4 (0.78–2.6)	1.4 (0.78–2.6)
Reedbuck	1.2 (0.94–1.5)	1.2 (0.94–1.5)	1.2 (0.94–1.5)	1.2 (0.94–1.5)	1.2 (0.94–1.5)	1.2 (0.94–1.5)
Small canids	2.8 (2.0–4.0)	2.8 (2.0–4.0)	2.8 (2.0–4.0)	2.8 (2.0–4.0)	2.8 (2.0–4.0)	2.8 (2.0–4.0)
Thomson's gazelle	15.7 (11.9–20.8)	16.1 (12.1–21.4)	31.9 (23.1–43.9)	9.2 (6.4–13.2)	6.7 (3.7–12.1)	4.1 (2.6–6.6)
Topi	2.3 (1.6–3.3)	5.7 (4.5–7.3)	3.9 (2.8–5.5)	0.85 (0.49–1.5)	0.93 (0.6–1.5)	0.55 (0.0002–140.6)
Warthog	5 (3.6–6.8)	5.7 (4.3–7.7)	11.1 (8.0–15.3)	5.9 (4.3–8.0)	3.1 (2.2–4.5)	7 (5.1–9.5)
Wildebeest	14.5 (4.3–48.9)	4 (1.9–8.3)	9.9 (5.5–18.0)	4.1 (1.5–11.5)	23.2 (15.8–33.9)	0.99 (0.11–8.8)
Zebra	10.4 (5.2–20.8)	8.9 (6.9–11.4)	18.6 (12.8–27.2)	10.3 (6.6–16.2)	12 (9.4–15.4)	16.2 (6.6–40.0)

Density of wildlife species most commonly sighted in transects, per km^2^, with 95% confidence intervals in parentheses.

**Table 4:** Host species composition at different study sites.

Study site	1		2		3		4		5		6	
	d	%	d	%	d	%	d	%	d	%	d	%
Baboon	2.6	3.3	2.6	2.6	2.6	1.7	2.6	2.8	2.6	2.1	2.6	1.4
Buffalo	7.1	8.9	9.1	9.2	4.4	2.8	7.1	7.5	8.1	6.5	10.9	5.7
Dikdik	0.0	0.0	1.8	1.8	2.0	1.3	3.2	3.4	4.6	3.7	15.3	8.0
Eland	0.2	0.3	0.2	0.2	0.2	0.1	0.2	0.2	0.2	0.2	0.2	0.1
Elephant	2.4	3.1	2.4	2.5	2.4	1.6	2.4	2.6	2.4	1.9	2.4	1.3
Giraffe	2.5	3.1	3.1	3.1	4.7	3.0	2.7	2.9	6.3	5.0	5.1	2.7
Grant's gazelle	2.4	3.0	4.2	4.2	2.7	1.7	0.1	0.1	0.3	0.2	0.0	0.0
Hartebeest	1.5	1.9	5.4	5.5	7.0	4.5	3.4	3.6	1.1	0.9	0.2	0.1
Hippopotamus	0.2	0.3	0.2	0.2	0.2	0.1	0.2	0.2	0.2	0.2	36.0	18.9
Hyaena	5.4	6.8	1.5	1.5	1.5	1.0	1.1	1.2	0.3	0.2	0.9	0.5
Impala	1.8	2.3	22.5	22.8	47.6	30.5	35.7	37.8	47.4	38.0	83.0	43.5
Large felids	1.4	1.8	1.4	1.4	1.4	0.9	1.4	1.5	1.4	1.1	1.4	0.7
Reedbuck	1.2	1.5	1.2	1.2	1.2	0.8	1.2	1.3	1.2	1.0	1.2	0.6
Small canids	2.8	3.5	2.8	2.8	2.8	1.8	2.8	3.0	2.8	2.2	2.8	1.5
Thomson's gazelle	15.7	19.8	16.1	16.3	31.9	20.4	9.2	9.7	6.7	5.4	4.1	2.1
Topi	2.3	2.9	5.7	5.8	3.9	2.5	0.9	0.9	0.9	0.7	0.6	0.3
Warthog	5.0	6.3	5.7	5.8	11.1	7.1	5.9	6.2	3.1	2.5	7.0	3.7
Wildebeest	14.5	18.3	4.0	4.0	9.9	6.3	4.1	4.3	23.2	18.6	1.0	0.5
Zebra	10.4	13.1	8.9	9.0	18.6	11.9	10.3	10.9	12.0	9.6	16.2	8.5
**Total**	79.5		98.9		156.2		94.5		124.9		190.9	

The density (d) of host species per square km, and proportion (%) out of total density of all species assessed at six study sites. Denser colour indicates higher density.

Two species were identified as bloodmeal sources but not observed on transects in sufficient numbers to give a density estimate: hippopotamus and eland. The value of the lowest density identified (0.2 animals/km^2^) was therefore assigned to hippopotamus and eland. For hippopotamus, one study site was known to have a high density of hippopotamus due to its proximity to a large pool. The hippopotamus density was not reflected in the distance data since hippopotamus are nocturnal, and transects were only conducted by day. The density of hippopotamus in rivers in nearby Maasai Mara was therefore used for this study site [42].

### Host feeding preference

**Fig 3:**
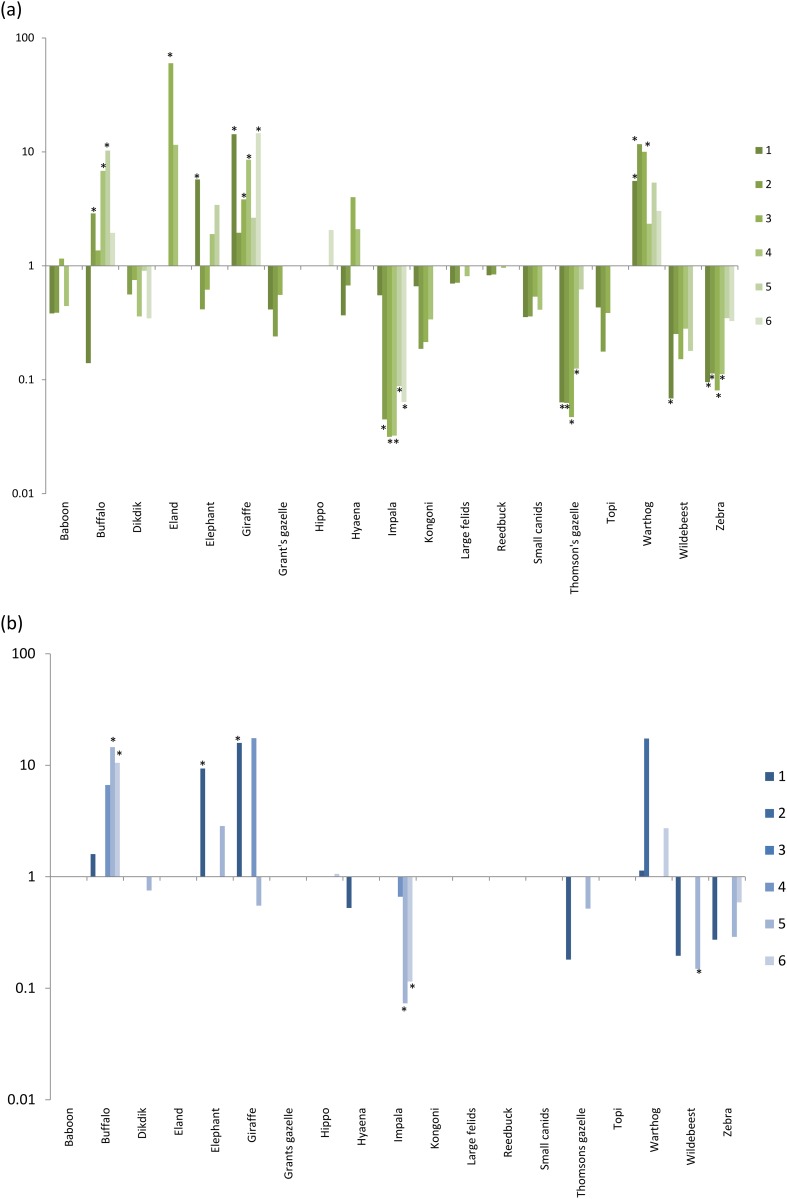
Feeding indices illustrate selection or avoidance of host species by Glossina swynnertoni and G. pallidipes. Feeding indices by species and study site on a log scale. Graded colours show the feeding index at each of 6 study sites. Values above 1 indicate a host is selected, values below 1 indicate a host is avoided, by (a) *G*. *swynnertoni* and (b) *G*. *pallidipes*. Stars indicate significance at p<0.05.

For *G*. *swynnertoni*, bloodmeals were taken preferentially from *w*arthog and giraffe in every study site (statistically significant at p<0.05 in 3/6 and 4/6 study sites respectively). Buffalo, elephant and eland were also significantly more frequently fed on in some study sites. A number of species were never identified in bloodmeals, despite comprising a large proportion of the wildlife hosts available, particularly impala, Thomson's gazelle, zebra and wildebeest (statistically significant in 5/6, 4/6, 4/6 and 1/6 study sites respectively, Fig 3a). For *G*. *pallidipes*, buffalo, elephant and giraffe were significantly more frequently fedon in at least one study site. Impala, Thomson’s gazelle and wildebeest were not fed on (Fig 3b).

## Discussion

This is the first study to have quantified the host contact rates of tsetse by combining molecular analysis of bloodmeals with robust contemporaneous measures of host species density. Identification of tsetse hosts across six study sites in Serengeti National Park, Tanzania, exhibiting local differences in wildlife species composition, allowed quantification of the degree of host selection and avoidance of *G*. *swynnertoni* and *G*. *pallidipes*, which is a key factor in determining the transmission potential of the host community.

Analyses of bloodmeals through matching of cytochrome B sequences successfully identified host species in 72% of samples from *G*. *swynnertoni* and 52% of samples from *G*. *pallidipes*. A major disadvantage of serological techniques previously used (for example [14]) was the need to raise antisera from putative host species. Sequence-based approaches avoid this issue and can identify any species for which reference sequences are available in Genbank. In this study sequences were available for the great majority of mammalian species likely to be present. Blood meal sequences that did not find close matches on BLAST searches could still be identified in many cases by alignment with available sequences from the species likely to be present. This, as well as the fact that two sequences that could not be identified were both poor quality sequences, suggested that non-identification was more likely to occur due to poor sequence quality than systematic non-identification of particular species.

Direct comparison of the rates of identification using sequence-based approaches cannot be made due to the lack of information within the academic literature on similar studies in tsetse. However, the proportion of samples successfully identified in our study is reasonably consistent with similar sequence-based techniques in different host vector systems for *G*. *swynnertoni*, but somewhat low for *G*. *pallidipes*. Successsful identifications were reported for 85% of bloodmeals from *Culicoides* species [43] and hosts were identified in 70% of mosquito bloodmeals [40]. Potential reasons for non-amplification include a lack of sufficient genetic material and failure of primers to recognise and amplify host material in tsetse flies. Bloodmeals taken more than 33 hours before sampling do not present a good template for DNA amplification [44]. Therefore the absence of target material may be due to either a small bloodmeal sample, or an extended time period between ingestion and sampling leading to degradation of the genetic material in the bloodmeal during the digestion process. In addition, use of FTA cards for preserving blood meal samples has not been assessed and may affect identification success, compared to preservation methods reported in other studies (for example DNA extraction from fresh tissue as in [45]). Reasons for lower identification success in *G*. *pallidipes* compared to *G*. *swynnertoni* are not clear. Failure to identify *G*. *pallidipes* samples occurred due to both failure to amplify, and generation of sequences of insufficient length or quality to be aligned. The wide availability of reference sequences for potential hosts makes it unlikely that *G*. *pallidipes* feed on a species that was not identified. There may be behavioural differences in *G*. *pallidipes*, for example to do with feeding intervals or blood meal size, that affect the likelihood of successful identification.

Double peaks were observed on some sequence chromatograms. These double peaks are likely to be associated with the presence of DNA from multiple hosts; this observation has been made previously [40,43,46]. These may represent the genetic remains of a previous bloodmeal (which could be 2–3 days old or longer), or interrupted feeding on more than one host species. In the future, use of tagged amplicon next generation sequencing approaches could allow better identification of multiple hosts [47].

The hosts most commonly identified from *G*. *swynnertoni* in this study are consistent with those found in the most recent large scale study in SNP in 1970, which used a haemaglutination method [20]. In this study, buffalo (27%), warthog (26%), giraffe (12%) and elephant (6%) were identified as the most important hosts [20]. In addition a wide range of hosts were identified that were found infrequently [20]. The relative importance of each species differs somewhat in our dataset, which may reflect changes in wildlife population size over the last 50 years; for example the buffalo population in the Serengeti ecosystem was estimated at 25000 in 2009 [48] compared to over 60000 in 1970s [49]. Our study found that although *G*. *swynnertoni* does have clear preferred hosts, they are also opportunistic feeders that can feed on a range of species. Tsetse have been identified feeding on birds [20,25,50] but most earlier studies, using serological techniques, identified meals simply as avian. The use of sequence data in this study allowed more accurate identification of species, with *G*. *swynnertoni* observed to have fed on kori bustard, ostrich and guinea fowl.

Bloodmeal feeding patterns have not previously been reported in SNP for *G*. *pallidipes* in large enough numbers to draw conclusions [20]. Studies in other ecosystems identified buffalo, bushbuck, warthog and bushpig to be the most important hosts for *G*. *pallidipes* [14,51]. Although both the number of samples collected and bloodmeals identified in this study were also small (46 sequences identified), they shed more light on the feeding patterns of *G*. *pallidipes*, with buffalo forming majority of meals (57%), and giraffe (20%) and elephant (11%) also commonly identified.

For *G*. *swynnertoni*, *w*arthog and giraffe were fed on preferentially in every study site, despite the relatively low density of these species. Warthog were fed on between two and 11 times more frequently than would be expected based on density; giraffe between two and 15 times more frequently. Elephant, eland and buffalo were also identified more often than would be expected (although it was not possible to estimate density of eland so this result should be interpreted with caution). *G*. *pallidipes* fed on buffalo, elephant and giraffe over other more common species. Neither *G*. *swynnertoni* nor *G*. *pallidipes* were found to feed on wildebeest, zebra, Thomson’s gazelle and impala, despite these four species being found at the highest densities when considered across the study sites. Other common antelope species, such as hartebeest, topi and Grant’s gazelle were also not identified in blood meals.

One aim of this study was to assess the correlation between wildlife abundances and tsetse feeding patterns. The differences between the measured density of wildlife species compared to the ‘abundance’ observed by tsetse when feeding could help to identify how tsetse feeding choices are made. A number of ecological, physical and behavioural mechanisms have been identified that influence host choice by tsetse. Savannah tsetse locate their hosts through a combination of long-range responses to host odours and short range responses to visual cues [52]. Host odours do not seem to influence feeding behaviour, as with the exception of human, odours from different host species appear to be equally attractive; although larger animals will produce comparatively greater doses of odour and hence attract more flies [18,19,53]. The probability of an attracted tsetse landing and feeding on a host is strongly related to the host’s defensive behaviour; feeding rates are reduced on animals that display high rates of skin rippling, kicking and tail-flicking in response to biting flies [54,55]. These defensive behaviours pose a risk to feeding tsetse and the avoidance of feeding on ‘risky’ hosts is thought to be an important driver of host selection behaviour [56]. Impala, despite their preference for wooded areas where tsetse are plentiful, display high rates of defensive behaviour (such as skin rippling), which prevents tsetse from feeding successfully [54]. Visual factors influencing attractiveness to tsetse have also been described, with the low feeding rate on zebra being ascribed by some authors to coloration [57–59]. Avoidance of tsetse habitat by particular wild hosts, or at times of day when tsetse are most active, could also be mechanisms for tsetse avoidance. However, this study assessed host density and tsetse feeding preferences in the same areas at a detailed level and at similar times of day, confirming that the host species considered were all found in areas where tsetse were abundant. The analysis in this study was also repeated using biomass (density x weight) instead of density alone. Qualitatively this did not change the pattern for any species other than elephant, which was then fed on less than expected for its biomass. Giraffe was fed on preferentially in 3 out of 6 sites. Warthog, buffalo, hyaena and eland were still fed on more than would be expected, and wildebeest, zebra, impala and Thomson’s gazelle less than would be expected. Although size is likely to play a role, for example by generating more odours, it is not sufficient to explain tsetse feeding behaviour. Aspects of tsetse feeding behaviour have been exploited differently by different host species, which defines the availability of hosts to tsetse. For example, it seems likely that other antelope species (gazelles, wildebeest) would also display the defensive behaviour which is thought to limit tsetse feeding on impala, whilst zebra rely more on visual mechanisms for avoidance. The species more commonly fed on (warthog, buffalo, giraffe, elephant) are not related taxonomically but perhaps share a tolerance to trypanosome infections that has reduced pressure for evolution of the various avoidance mechanisms relied by other species.

The second aim of this paper was to quantify tsetse-host contact rates to understand transmission of *T*. *b*. *rhodesiense*. *G*. *swynnertoni* were found to be feeding on warthog, buffalo and giraffe at significantly higher levels than would be expected from the density of these species. Warthog are known to carry a number of trypanosome species, including *T*. *b*. *rhodesiense* [7,60], with *T*. *b*. *rhodesiense* prevalence of 9.5% found in warthog in Serengeti [7].Their predominance as tsetse hosts combined with high prevalence of *T*. *b*. *rhodesiense* suggests they may be of key importance in *T*. *b*. *rhodesiense* transmission. *T*. *b*.*rhodesiense* was recently identified in buffalo [3] and the finding that both *G*. *swynnertoni* and *G*. *pallidipes* actively select buffalo to feed on suggests they may also be an important part of the transmission community. *T*. *brucei* s.l. has only rarely been identified in giraffe and elephant [7,61–63]. Since over 25% of *G*. *swynnertoni* bloodmeals are from giraffe and elephant, these species may be particularly important as a host species reducing transmission of *T*. *brucei* s.l., possibly acting as ‘dilution hosts’.

Of the species that were rarely fed on, *T*. *brucei* s.l. has been found in wildebeest, zebra and impala [7,62,64,65]. *T*. *brucei* is only transmitted via tsetse, indicating that although the host-vector contact rate is low, tsetse clearly do sometimes feed on these species.. It is known that fly behaviour changes with increasing time between feeds. Tsetse can be assumed to avoid feeding on some species when the risk of being killed by the host’s defensive behaviour is higher than the risk of starvation. As a fly’s nutritional reserves decline following a feed, the risk of starvation increases and the relative benefits of feeding on a ‘risky’ host increase. It is likely that the small numbers of feeds found to contain a range of less commonly fed on hosts occur in circumstances when tsetse are hungry and therefore feed less selectively [56]. Speculatively, being rarely fed on may correlate with higher and more detectable parasitaemia, compared to animals that are constantly exposed to trypanosomes and may be better at controlling infection. It is possible that these species, though rarely fed on, could still be important in driving transmission, particularly as hungry flies are also more susceptible to *T*. *brucei* infection [66]. Further data on the prevalence of *T*. *brucei* and *T*. *b*. *rhodesiense* in different hosts would be of value to further evaluate the roles of different wildlife species.

In summary, this study has quantified not only the feeding preferences but also the selection and avoidance of hosts by *G*. *swynnertoni* and *G*. *pallidipes* in an area of high host density and diversity. The extreme selectivity exhibited by these species, along with the ability of the key host species to maintain and transmit *T*. *b*. *rhodesiense*, drives the epidemiology of HAT in wilderness areas. This study highlights the importance of measuring host density when assessing vector feeding patterns, allowing increased understanding of drivers for both selection and avoidance by vectors, as well as providing important parameters for modelling system dynamics.

## Supporting Information

S1 Table List of species included in sequence database where sequences available, with accession numbers. (DOCX)

S2 Table Genbank accession numbers for the blood meal samples that were matched to >97% in BLAST searches. (DOCX)

## References

[1] Welburn SC, Picozzi K, Fevre EM, Coleman PG, Odiit M, et al (2001) Identification of human-infective trypanosomes in animal reservoir of sleeping sickness in Uganda by means of serum- resistance-associated (SRA) gene. Lancet 358: 2017–2019. 11755607 10.1016/s0140-6736(01)07096-9

[2] Welburn SC, Picozzi K, Fyfe J, Fèvre E, Odiit M, et al (2005) Control Options for Human Sleeping Sickness in Relation to the Animal Reservoir of Disease In: Osofsky SA, Cleaveland S, Karesh WB, Kock MD, Nyhus PJ, et al, editors. Conservation and Development Interventions at the Wildlife/Livestock Interface: Implications for Wildlife, Livestock and Human Health. IUCN, Gland, Switzerland and Cambridge, UK pp. 55–61.

[3] Anderson NE, Mubanga J, Fevre EM, Picozzi K, Eisler MC, et al (2011) Characterisation of the wildlife reservoir community for human and animal trypanosomiasis in the Luangwa Valley, Zambia. PLoS Negl Trop Dis 5: e1211 10.1371/journal.pntd.0001211 21713019 PMC3119639

[4] Geigy R, Mwambu PM, Kauffman M (1971) Sleeping sickness survey in Musoma District, Tanzania: IV. Examination of wild mammals as a potential reservoir for T. rhodesiense. Acta Trop 28: 211–220. 4400764

[5] Geigy R, Kauffman M (1973) Sleeping sickness survey in the Serengeti area (Tanzania) 1971: I. Examination of large mammals for trypanosomes. Acta Trop 30: 12–23. 4144952

[6] Geigy R, Kauffman M, Mayende JSP, Mwambu PM, Onyango RJ (1973) Isolation of Trypanosoma (Trypanozoon) rhodesiense from game and domestic animals in Musoma District, Tanzania. Acta Trop 30: 50–56. 4144957

[7] Kaare MT, Picozzi K, Mlengeya T, Fevre EM, Mellau LS, et al (2007) Sleeping sickness—a re-emerging disease in the Serengeti? Travel Med Infect Dis 5: 117–124. 17298919 10.1016/j.tmaid.2006.01.014

[8] Heisch RB, McMahon JP, Mansonbahr PEC (1958) The isolation of Trypanosoma rhodesiense from a bushbuck. Br Med J 2: 1203–1204. 13584899 10.1136/bmj.2.5106.1203 PMC2027234

[9] Robson J, Rickman LR, Scott D, Allsopp R (1972) Composition of Trypanosoma brucei subgroup in nonhuman reservoirs in Lambwe-Valley, Kenya, with particular reference to distribution of T. rhodesiense. Bull World Health Organ 46: 765–770. 4538538 PMC2480874

[10] Welburn SC, Picozzi K, Coleman PG, Packer C (2008) Patterns in age-seroprevalence consistent with acquired immunity against *Trypanosoma brucei* in Serengeti lions. PLoS Negl Trop Dis 2: e347 10.1371/journal.pntd.0000347 19065258 PMC2586656

[11] Simarro PP, Diarra A, Postigo JAR, Franco JR, Jannin JG (2011) The Human African Trypanosomiasis Control and Surveillance Programme of the World Health Organization 2000–2009: The Way Forward. PLoS Negl Trop Dis 5 e1007 10.1371/journal.pntd.0001007 21364972 PMC3042999

[12] Simpson JE, Hurtado PJ, Medlock J, Molaei G, Andreadis TG, et al (2012) Vector host-feeding preferences drive transmission of multi-host pathogens: West Nile virus as a model system. Proc Biol Sci 279: 925–933. 10.1098/rspb.2011.1282 21849315 PMC3259921

[13] Kilpatrick AM, Daszak P, Jones MJ, Marra PP, Kramer LD (2006) Host heterogeneity dominates West Nile virus transmission. Proc R Soc B-Biological Sci 273: 2327–2333. 10.1098/rspb.2006.3575 PMC1636093 16928635

[14] Clausen P-H, Adeyemi I, Bauer B, Breloeer M, Salchow F, et al (1998) Host preferences of tsetse (Diptera:Glossinidae) based on bloodmeal identifications. Med Vet Entomol 12: 169–180. 9622371 10.1046/j.1365-2915.1998.00097.x

[15] Staak C, Kampe U, Korkowski G (1986) Species identification of blood-meals from tsetse flies (Glossinidae): results 1979–1985. Trop Med Parasitol 37: 59–60. 3704476

[16] Lamprey HF, Glasgow JP, Lee-jones F, Weitz B (1962) A simultaneous census of the potential and actual food sources of the tsetse fly Glossina swynnertoni Austen. J Anim Ecol 31: 151–156.

[17] LoGiudice K, Ostfeld RS, Schmidt K, Keesing F (2003) The ecology of infectious disease: effects of host diversity and community composition on Lyme disease risk. Proc Natl Acad Sci U S A 100: 567–571. Available: http://www.pubmedcentral.nih.gov/articlerender.fcgi?artid=141036&tool=pmcentrez&rendertype=abstract. Accessed 19 August 2013. 12525705 10.1073/pnas.0233733100 PMC141036

[18] Hargrove JW, Vale GA (1978) The effect of host odour concentration on catches of tsetse flies (Glossinidae) and other Diptera in the field. Bull Entomol Res 68: 607–612.

[19] Vale GA (1974) Responses of tsetse flies (Diptera, Glossinidae) to mobile and stationary baits. Bull Entomol Res 64: 545–588.

[20] Moloo SK, Steiger RF, Brun R, Boreham PFL (1971) Sleeping sickness survey In Musoma District, Tanzania: II. The role of Glossina in the transmission of sleeping sickness. Acta Trop 28: 189–205. 4400762

[21] Auty HK, Picozzi K, Malele II, Torr SJ, Cleaveland S, et al (2012) Using molecular data for epidemiological inference: assessing the prevalence of Trypanosoma brucei rhodesiense in tsetse in Serengeti, Tanzania. PLoS Negl Trop Dis 6: e1501 10.1371/journal.pntd.0001501 22303496 PMC3269424

[22] Fairbairn H (1948) Sleeping sickness in Tanganyika territory, 1922–1946. Trop Dis Bull 45: 1–17.

[23] Jelinek T, Bisoffi Z, Bonazzi L, van Thiel P, Bronner U, et al (2002) Cluster of African trypanosomiasis in travellers to Tanzanian national parks. Emerg Infect Dis 8: 634–635. 12023923 10.3201/eid0806.010432 PMC2738477

[24] Ripamonti D, Massari M, Arici C, Gabbi E, Farina C, et al (2002) African sleeping sickness in tourists returning from Tanzania: The first 2 Italian cases from a small outbreak among European travelers. Clin Infect Dis 34: E18–E22. 11731969 10.1086/338157

[25] Rogers DJ, Boreham PFL (1973) . Acta Trop 30: 24–35. 4144955

[26] Muturi CN, Ouma JO, Malele II, Ngure RM, Rutto JJ, et al (2011) Tracking the feeding patterns of tsetse flies (*Glossina* genus) by analysis of bloodmeals using mitochondrial cytochromes genes. PLoS One 6: e17284 10.1371/journal.pone.0017284 21386971 PMC3046180

[27] Hargrove JW, Langley PA (1990) Sterilizing tsetse (Diptera, Glossinidae) in the field—a successful trial. Bull Entomol Res 80: 397–403.

[28] Torr SJ, Hall DR, Phelps RJ, Vale GA (1997) Methods for dispensing odour attractants for tsetse flies (Diptera:Glossinidae). Bull Entomol Res 87: 299–311.

[29] Thomas L, Laake JL, Rexstad E, Strindberg S, Marques FFC, et al. (2009) Distance 6.0, release 2.

[30] Ogutu JO, Bhola N, Piepho H-P, Reid R (2006) Efficiency of strip- and line-transect surveys of African savanna mammals. J Zool 269: 149–160. 10.1111/j.1469-7998.2006.00055.x

[31] Sinclair ARE, Norton-Griffiths M (1979) Serengeti: Dynamics of an ecosystem. Chicago: University of Chicago Press.

[32] Buckland ST, Anderson DR, Burnham KP, Laake JL, Borchers DL, et al (2001) Introduction to Distance Sampling. Oxford: Oxford University Press.

[33] Ahmed HA, MacLeod ET, Hide G, Welburn SC, Picozzi K (2011) The best practice for preparation of samples from FTA cards for diagnosis of blood borne infections using African trypanosomes as a model system. Parasites and Vectors 4: 68 10.1186/1756-3305-4-68 21548975 PMC3108913

[34] Hall TA (1999) BioEdit: a user-friendly biological sequence alignment editor and analysis program for Windows 95/98/NT. Nucleic Acids Symp Ser 41: 95–98.

[35] Mduma SAR, Hopcraft JGC (2008) The main herbivorous mammals and crocodiles in the greater Serengeti ecosystem In: Sinclair ARE, Packer C, Mduma S, Fryxell J, editors. Serengeti III Human impacts on ecosystem dynamics. Chicago: University of Chicago Press.

[36] Thompson JD, Higgins DG, Gibson TJ (1994) Clustal-W-Improving the sensitivity of progressive multiple sequence alignment through sequence weighting, position-specific gap penalties and weight matrix choice. Nucleic Acids Res 22: 4673–4680. 7984417 10.1093/nar/22.22.4673 PMC308517

[37] Drummond AJ, Ashton B, Buxton S, Cheung M, Cooper A, et al. (2010) Genious v5.3 Available: http://www.geneious.com.

[38] Hasegawa M, Kishino H, Yano TA (1985) Dating of the human ape splitting by a molecular clock of mitochondrial DNA. J Mol Evol 22: 160–174. 3934395 10.1007/BF02101694

[39] Lardeux F, Loayza P, Bouchité B, Chavez T (2007) Host choice and human blood index of Anopheles pseudopunctipennis in a village of the Andean valleys of Bolivia. Malar J 6: 8 10.1186/1475-2875-6-8 17241459 PMC1783659

[40] Hamer GL, Kitron UD, Goldberg TL, Brawn JD, Loss SR, et al (2009) Host selection by Culex pipiens mosquitoes and West Nile virus amplification. Am J Trop Med Hyg 80: 268–278. 19190226

[41] Hassan HK, Cupp EW, Hill GE, Katholi CR, Klingler K, et al (2003) Avian host preference by vectors of eastern equine encephalomyelitis virus. Am J Trop Med Hyg 69: 641–647. 14740882

[42] Kanga EM, Ogutu JO, Olff H, Santema P (2011) Population trend and distribution of the Vulnerable common hippopotamus Hippopotamus amphibius in the Mara Region of Kenya. Oryx 45: 20–27. 10.1017/S0030605310000931

[43] Calvo JH, Berzal B, Calvete C, Miranda M, Estrada R, et al (2012) Host feeding patterns of Culicoides species (Diptera: Ceratopogonidae) within the Picos de Europa National Park in northern Spain. Bull Entomol Res 102: 692–697. 10.1017/S0007485312000284 22647415

[44] Oshaghi MA, Chavshin AR, Vatandoost H, Yaaghoobi F, Mohtarami F, et al (2006) Effects of post-ingestion and physical conditions on PCR amplification of host blood meal DNA in mosquitoes. Exp Parasitol 112: 232–236. 10.1016/j.exppara.2005.11.008 16364301

[45] Hamer GL, Kitron UD, Goldberg TL, Brawn JD, Loss SR, et al (2009) Host selection by Culex pipiens mosquitoes and west nile virus amplification. Am J Trop Med Hyg 80: 268–278. 80/2/268 [pii]. 19190226

[46] Alcaide M, Rico C, Ruiz S, Soriguer R, Muñoz J, et al (2009) Disentangling vector-borne transmission networks: a universal DNA barcoding method to identify vertebrate hosts from arthropod bloodmeals. PLoS One 4: e7092 10.1371/journal.pone.0007092 19768113 PMC2740869

[47] Meier R, Wong W, Srivathsan A, Foo M (2016) $1 DNA barcodes for reconstructing complex phenomes and finding rare species in specimen-rich samples. Cladistics 32: 100–110. 10.1111/cla.12115 34732017

[48] Tanzania Wildlife Research Institute (2010) Aerial census in the Serengeti ecosystem, wet season 2010.

[49] Sinclair ARE, Mduma SAR, Hopcraft JGC, Fryxell JM, Hilborn R, et al (2007) Long-term ecosystem dynamics in the Serengeti: lessons for conservation. Conserv Biol 21: 580–590. 10.1111/j.1523-1739.2007.00699.x 17531037

[50] Weitz B (1963) The feeding habits of Glossina. Bull World Health Organ 28: 711–729. 13999790 PMC2554947

[51] Glasgow JP, Isherwood F, Lee-Jones F, Weitz B (1958) Factors influencing the staple food of tsetse. J Anim Ec 27: 59–69.

[52] Gibson G, Torr SJ (1999) Visual and olfactory responses of haematophagous Diptera to host stimuli. Med Vet Entomol 13: 2–23. 10.1046/j.1365-2915.1999.00163.x 10194745

[53] Hargrove JW, Holloway MTP, Vale GA, Gough AJE, Hall DR (1995) Catches of tsetse (Glossina spp.) (Diptera: Glossinidae) from traps and targets baited with large doses of natural and synthetic host odour. Bull Entomol Res 85: 215 10.1017/S0007485300034295

[54] Vale GA (1977) Feeding responses of tsetse flies (Diptera: Glossinidae) to stationary hosts. Bull Entomol Res 67: 635–649.

[55] Torr SJ (1994) Responses of tsetse flies (Diptera: Glossinidae) to warthog (Phacochoerus aethiopicus Pallas). Bull Entomol Res 84: 411–419. 10.1017/S0007485300032545

[56] Hargrove JW, Williams BG (1995) A cost-benefit analysis of feeding in female tsetse. Med Vet Entomol 9: 109–119. 7787217 10.1111/j.1365-2915.1995.tb00166.x

[57] Gibson G (1992) Do tsetse flies “see” zebras? A field study of the visual response of tsetse to striped targets. Physiol Entomol 17: 141–147. 10.1111/j.1365-3032.1992.tb01191.x

[58] Caro T, Izzo A, Reiner RC, Walker H, Stankowich T (2014) The function of zebra stripes. Nat Commun 5: 3535 10.1038/ncomms4535 24691390

[59] Waage JK (1981) How the zebra got its stripes—biting flies as selective agents in the evolution of zebra coloration. J Entomol Soc South Afr 44: 351–358.

[60] Awan MAQ (1979) Identification by the Blood Incubation Infectivity Test of Trypanosoma brucei subspecies isolated from game animals in the Luangwa Valley, Zambia. Acta Trop 36: 343–347. 44098

[61] Vanderplank FL (1947) Seasonal and annual variation in the incidence of trypanosomiasis in game. Annu Trop Med Parasitol 41: 365–374. 10.1080/00034983.1947.11685339 18902135

[62] Baker JR (1968) Trypanosomes of wild mammals in the neighbourhood of the Serengeti National Park. Symp Zool Soc London 24: 147–158.

[63] Dillmann JSS, Townsend AJ (1979) Trypanosomiasis survey of wild animals in the Luangwa Valley, Zambia. Acta Trop 36: 349–356. 44099

[64] Irvin AD, Omwoyo P, Purnell RE, Peirce MA, Schieman B (1973) Blood parasites of impala (Aepyceros melampus) in Serengeti National Park. Vet Rec 93: 200–203. 4202696 10.1136/vr.93.7.200

[65] Auty HK, Anderson NE, Picozzi K, Lembo T, Mubanga J, et al (2012) Trypanosome diversity in wildlife species from the serengeti and Luangwa Valley ecosystems. PLoS Negl Trop Dis 6: e1828 10.1371/journal.pntd.0001828 23094115 PMC3475651

[66] Kubi C, Van den Abbeele J, De Deken R, Marcotty T, Dorny P, et al (2006) The effect of starvation on the susceptibility of teneral and non-teneral tsetse flies to trypanosome infection. Med Vet Entomol 20: 388–392. 17199750 10.1111/j.1365-2915.2006.00644.x

