# Supplementary material for: Quantifying Heterogeneity in Host-Vector Contact: Tsetse (Glossina swynnertoni and G. pallidipes) Host Choice in Serengeti National Park, Tanzania
Source: PLoS One. 2016 Oct 5;11(10):e0161291. doi: 10.1371/journal.pone.0161291 (PMC5051720; doi:10.1371/journal.pone.0161291)
Supplement: S1 Table — (DOCX) [file pone.0161291.s001.docx]

| **Table S1**  **List of species included in sequence database where sequences available, with accession numbers** | | |
| --- | --- | --- |
| **Common name** | **Scientific name** | **Sequence used** |
| Four-toed hedgehog | *Atelerix albiventris* | KF7831431 |
| Black and white colobus | *Colobus guereza* | U38264 |
| Olive baboon | *Papio anubis* | EU885459 |
| Patas monkey | *Erythocebus patas* | NC_021947 |
| Vervet monkey | *Chlorocebus pygerythrus* | JX983851 |
| Greater galago | *Otolemur crassicaudatus* | AY441465 |
| Bushbaby lesser | *Galago senegalensis* | KP410636 |
| Lion | *Panthera leo* | AF384817 |
| Spotted hyaena | *Crocuta crocuta* | AF511064 |
| Cheetah | *Acinonyx jubatus* | NC_005212 |
| Leopard | *Panthera pardalis* | JF720185 |
| African wild dog | *Lycaon picuts* | KT447689 |
| Black-backed jackal | *Canis mesomelas* | KT447688 |
| Golden jackal | *Canis aureus* | KT447747 |
| Side-striped jackal | *Canis adustus* | KT447687 |
| Bat-eared fox | *Otocyon megalotis* | Alignment not possible |
| Striped hyaena | *Hyaena hyaena* | AY928678 |
| Aardwolf | *Proteles cristatus* | AY928679 |
| Wildcat | *Felis silvestris* | EF689046 |
| Serval | *Leptailurus serval* | NC_028316 |
| Caracal | *Caracal caracal* | NC_028306 |
| Egyptian mongoose | *Herpestes ichneumon* | AF5110591 |
| Banded mongoose | *Mungos mungo* | AY928674 |
| Dwarf mongoose | *Helogale parvula* | AF522334 |
| Black-tipped mongoose | *Galerella sanguinea* | Alignment not possible |
| Genet, common | *Genetta genetta* | AY241922 |
| White tailed mongoose | *Ichneumia albicauda* | AF511058 |
| Marsh mongoose | *Atalix paludinosus* | AF522325 |
| Civet, African | *Civettictis civetta* | AF511043 |
| Honey badger | *Mellivora capensis* | EF987755 |
| Zorilla | *Ictonyx striatus* | AF498156 |
| Palm civet | *Nandinia binotata* | AF511057 |
| Striped weasel African | *Poecilogale albinucha* | EF472349 |
| Spotted necked otter | *Lutra maculicollis* | AF057125 |
| Cape clawless otter | *Aonyx capensis* | AF057118 |
| Elephant | *Loxodonta africana* | AY742801 |
| Bush hyrax | *Heterohyrax brucei* | AF045137 (not full length) |
| Rock hyrax | *Procavia capensis* | D86909 |
| Tree hyrax | *Dendrohyrax arboreus* | Not available, AM904727 Western Tree Hyrax Dendrohyrax dorsalis used instead. |
| Aardvark | *Orycteropus afer* | AF107724 |
| Zebra burchell’s | *Equus burchelli* | JF718888 |
| Rhinoceros, black | *Diceros bicornis* | JF718876 |
| Giraffe | *Giraffa camelopardalis* | AY534342 |
| Buffalo, African | *Syncerus caffer* | AF036275 |
| Eland | *Tragelaphus oryx* | AF022057 |
| Topi | *Damaliscus lunatus* | AF016635 |
| Kongoni | *Alcelaphus buselaphus* | JN632593 |
| Wildebeest | *Connochaetus taurinus* | AF016638 |
| Impala | *Aepyceros melampus* | AF036289 |
| Defassa waterbusk | *Kobus defassa* | AF096625 |
| Bohor reedbuck | *Redunca redunca* | AF096626 |
| Mountain reedbuck | *Redunca fulvorufula* | AF036284 |
| Thomson’s gazelle | *Gazelle thomsoni* | DQ470795 |
| Grant’s gazelle | *Gazella granti* | AF028820. |
| Oryx | *Oryx beisa* | DQ138192 |
| Roan antelope | *Hippotragus equinus* | HQ641316 |
| Oribi | *Ourebia ourebi* | AF320574 |
| Klipspringer | *Oreotragus oreotragus* | AF036288 |
| Dikdik, Kirk’s | *Madoqua kirkii* | JF489137 |
| Steinbuck | *Raphicerus campestris* | AF022068 |
| Hippopotamus | *Hippopotamus amphibius* | U07565 |
| Greater kudu | *Tragelaphus strepsiceros* | HQ641313 |
| Lesser kudu | *Tragelaphus imberbis* | AF036279 |
| Bushbuck | *Tragelaphus scriptus* | AF036277 |
| Warthog | *Phacochoerus africanus* | AJ314547 |
| Bushpig | *Potamochoerus porcus* | AY534300 |
| Giant forest hog | *Hylochoerus meinertzhageni* | GQ338968 |
| Pangolin, ground | *Manis temminckii* | KP306516 |
| Spring hare | *Pedetes capensis* | U59177 |
| Cape crested porcupine | *Hystrix africaeaustralis* | X70674 |
| North African crested porcupine | *Hystrix cristata* | FJ472574 |
| Cape hare | *Lepus capensis* | AJ279415 |
| Crawshay’s hare | *Lepus crawshayi* | Not available |
| Red rock hare | *Pronolagus rupestris* | AY292735 |
| African soft furred rat | *Mastomys natalensis* | EU914106 |
| African grass rat | *Arvicanthis niloticus* | KF478426 |
| Kaiser’s rock rat | *Aethomys kaiseri* | AJ604520 |
| African pygmy mouse | *Mus minutoides* | AJ875081 |
| Nile crocodile | *Crocodylus niloticus* | JF315273 |
| Nile monitor lizard | *Varanus niloticus* | NC_008778 |
| Rock python | *Python sebae* | U69863 |
| Puff adder | *Bitis arietans* | KJ415299 (not full length) |
| Kori bustard | *Ardeotis kori* | AJ511440 |
| Helmeted guinea fowl | *Numida meleagris* | L08383 |
| Ostrich | *Struthio camelus* | U76055 |
