# Supplementary material for: Quantifying Heterogeneity in Host-Vector Contact: Tsetse (Glossina swynnertoni and G. pallidipes) Host Choice in Serengeti National Park, Tanzania
Source: PLoS One. 2016 Oct 5;11(10):e0161291. doi: 10.1371/journal.pone.0161291 (PMC5051720; doi:10.1371/journal.pone.0161291)
Supplement: S2 Table — (DOCX) [file pone.0161291.s002.docx]

S2. Table 1. Genbank accession numbers for the blood meal samples that were matched to >97% in BLAST searches.

| KX697341 | KX697342 | KX697343 | KX697344 | KX697345 | KX697346 |
| --- | --- | --- | --- | --- | --- |
| KX697347 | KX697348 | KX697349 | KX697350 | KX697351 | KX697352 |
| KX697353 | KX697354 | KX697355 | KX697356 | KX697357 | KX697358 |
| KX697359 | KX697360 | KX697361 | KX697362 | KX697363 | KX697364 |
| KX697365 | KX697366 | KX697367 | KX697368 | KX697369 | KX697370 |
